# Zinc Ions Inactivate Influenza Virus Hemagglutinin and Prevent Receptor Binding

**DOI:** 10.3390/biomedicines13081843

**Published:** 2025-07-29

**Authors:** Ahn Young Jeong, Vikram Gopal, Aartjan J. W. te Velthuis

**Affiliations:** 1 Lewis Thomas Laboratory, Department of Molecular Biology, Princeton University, Princeton, NJ 08544, USA; aj3811@princeton.edu; 2 Ascend Performance Materials, 1010 Travis Street, Suite 900, Houston, TX 77002, USA; vgopal@ascendmaterials.com

**Keywords:** influenza A virus, hemagglutinin, zinc, face mask, PPE

## Abstract

**Background:** Influenza A viruses (IAV) cause seasonal flu and occasional pandemics. In addition, the potential for the emergence of new strains presents unknown challenges for public health. Face masks and other personal protective equipment (PPE) can act as barriers that prevent the spread of these viruses. Metal ions embedded into PPE have been demonstrated to inactivate respiratory viruses, but the underlying mechanism of inactivation and potential for resistance is presently not well understood. **Methods:** In this study, we used hemagglutination assays to quantify the effect of zinc ions on IAV sialic acid receptor binding. We varied the zinc concentration, incubation time, incubation temperature, and passaged IAV in the presence of zinc ions to investigate if resistance to zinc ions could evolve. **Results:** We found that zinc ions impact the ability of IAV particles to hemagglutinate and observed inhibition within 1 min of exposure. Maximum inhibition was achieved within 1 h and sustained for at least 24 h in a concentration-dependent manner. Inhibition was also temperature-dependent, and optimal above room temperature. Serial passaging of IAV in the presence of zinc ions did not result in resistance. **Conclusions:** e conclude that zinc ions prevent IAV hemagglutination in a concentration and temperature-dependent manner for at least 24 h. Overall, these findings are in line with previous observations indicating that zinc-embedded materials can inactivate the IAV hemagglutinin and SARS-CoV-2 spike proteins, and they support work toward developing robust, passive, self-cleaning antiviral barriers in PPE.

## 1. Introduction

Emerging infectious agents can have a devastating impact on public health and the global economy [1]. Among various emerging infectious agents, RNA viruses are responsible for several recent pandemics and epidemics. Recent and potential future examples include outbreaks of the 2009 pandemic influenza A virus (IAV) strain [2], SARS-CoV-2, the causative agent of COVID-19 [3], and Ebola virus [4]. The outbreak frequency of emerging and re-emerging influenza viruses has increased since the 1940s due to intensified human and animal transportation and extensive interactions between humans and the environment [5,6]. While various studies aim to predict or evaluate the risk associated with the next pandemic virus [7,8,9,10] and medical advances such as vaccines and antiviral therapeutics can reduce the mortality and symptoms associated with RNA virus infection [11,12,13], the development of personal protective equipment (PPE) with virus-inactivating properties may help limit the impact of future emerging RNA viruses.

Of particular interest as future pandemic RNA viruses are IAV strains [2,10]. Influenza viruses are the causative agent of flu, an acute respiratory disease with symptoms ranging from a mild fever and sore throat to lethal pneumonia and extra-respiratory complications [5,14]. In humans, flu is primarily caused by IAV and influenza B virus (IBV) strains [15,16], but some evidence for human influenza C virus infections exists [17]. Despite the availability of annual flu vaccinations and antiviral treatments [18], influenza remains a major burden to human health systems, with 3–5 million cases and up to 650,000 deaths reported every year [19]. IAVs have also been responsible for four major pandemics since the 1918 pandemic flu [2]. The emergence of these pandemic IAV strains, such as the H3N2, H2N2 and H1N1 strains in the last century, is partly driven by an antigenic shift [20,21,22,23]. This can occur when exchange or reassortment of the hemagglutinin (HA) encoding gene occurs between a seasonal and an avian IAV strain. Concerns about future flu pandemics thus focus on the increased circulation of avian influenza virus strains like H5N1, H5N8, and H7N9, and the ability of these HPAIVs to infect and cause disease in humans [24,25,26,27,28].

The start of an IAV infection depends on the molecular function of the HA protein. The HA protein resides on the outside of IAV particles and binds alpha-2,3 and/or alpha-2,6 sialic acid receptors [29]. Following the binding to the sialic acid, IAV particles are internalized into endosomes, where the acidic environment subsequently triggers a conformational rearrangement of the HA trimers, facilitating fusion between the viral and host membranes [30,31]. Membrane fusion starts the release of the viral ribonucleoprotein complexes for transport to the nucleus, where transcription and replication of the IAV genome occurs [32]. Antibodies generated upon vaccination or ion channel inhibitors taken orally may prevent different stages of the IAV infection cycle. An alternative strategy to vaccination or drug therapy is inactivation of an IAV particle, for instance through UV radiation, which creates crosslinks in the IAV RNA genome, or virus-inactivating PPE containing divalent ions [33,34,35,36].

PPE serve as a first line of defense against infectious pathogens, trapping virus particles, bacteria or fungal spores in a fibrous mesh. This may protect individuals or prevent the spread of a communicable pathogen. Individuals who frequently come into contact with poultry, cattle or wild animals should therefore use PPE and mitigate the risk of human infection and potential outbreaks. We and others have shown that metal ions embedded in nylons or sprays can be used to reduce the titer of viruses and bacteria [33,34,37,38,39]. Specifically, we contributed to the development of a reusable zinc ion-embedded polyamide 6.6 fiber (PA66; containing 0.065% w/w/zinc oxide) capable of inactivating IAV and SARS-CoV-2 [33]. However, the mechanism through which IAV and SARS-CoV-2 are inactivated by zinc ions is currently not fully understood and it is unclear if exposure to zinc ions can lead to resistance. A better understanding of the inactivation mechanism is critical to devise better validation assays and further develop self-cleaning PPE.

Here, we sought to determine whether zinc ions can alter the reception binding function of the IAV HA protein on intact virions using well-established hemagglutination assays. Previous work by Gopal et al. [33] demonstrated that zinc ions reduced IAV titers through a mechanism that does not involve releasing viral RNA, suggesting that the viral envelope remains intact and that the surface glycoprotein HA is the likely target of zinc ions. In addition, Seok et al. provided evidence that zinc and copper ions affect HA directly, with zinc binding specifically to Glu68 and His 137 in the HA1 subunit, and inducing conformational rearrangements that mimic the acidic pH-induced transition observed during endosomal fusion [40]. However, it is currently unknown whether such zinc-mediated structural alterations impair HA’s receptor binding function in the context of an intact virion.

We provide a bridge between the above two studies and demonstrate that exposure of IAV virions to zinc ions impedes IAV hemagglutination in a concentration- and time-dependent manner. Notably, this inhibition persists for 24 h after zinc ions are chelated with ethylenediaminetetraacetic acid (EDTA), suggesting that zinc ions induce a lasting structural change that prevents viral attachment and infection. Moreover, six serial passages of IAV in the presence of zinc ions did not result in resistance to zinc inhibition. Overall, our results together with the existing literature suggest that zinc ions cause HA structural alterations or HA aggregation and thereby inactivate in intact IAV virions. Our results also indicate that standard virology assays can be used to validate the inactivating properties of self-cleaning PPE.

## 2. Materials and Methods

### 2.1. Influenza Viruses and Cells

Madin-Darby canine kidney (MDCK) cells, originally sourced from American Type Culture Collection, were grown in Dulbecco’s Modified Eagle Medium (DMEM) (GeneDepot, Baker, CA, USA; Cat: CM014-050) supplemented with 10% fetal bovine serum (FBS; Life Technologies Corporation, Grand Island, NE, USA; Cat: 0540819), high glucose, pyruvate and glutamine. MDCK cells were used to amplify influenza A/WSN/33 (H1N1) virus in DMEM with 0.5% FBS at 37 °C and 5% CO_2_ or to titrate these viral stocks using plaque assays as described previously [33].

### 2.2. Zinc Incubation and Neutralization with EDTA

To assess the effects of zinc or copper ions on IAV, 100 µL of IAV strain A/WSN/33 (H1N1) (7.13 × 10^7^ pfu/mL) was incubated with 100 µL of 0, 1, 2, 5, or 10 mM zinc chloride (ZnCl_2_; Thermo Scientific, Heysham, UK; Cat: A16281.36) or copper chloride (Thermo Scientific, Ward Hill, MA, USA; Cat: 012457.18 working solutions prepared in DMEM containing 2% FBS at RT. After incubating the samples for the time periods indicated in the figures, or an hour if not indicated, zinc ions were chelated by adding equimolar EDTA (Lab Chem, Zelienople, PA, USA; Cat: LC137501), also prepared in DMEM supplemented with 2% FBS, to minimize cytotoxic effects on cell-based receptor binding or infection experiments.

### 2.3. Hemagglutination Assays

To evaluate the effects of zinc ions on sialic acid receptor binding abilities of IAVs, hemagglutination assays were performed using turkey red blood cells (RBCs; Lampire, Pipersvile, PA, USA; Cat: 7249407). Two-fold serial dilution of samples were prepared in a U-bottom 96-well microtiter plate (Perkin Elmer) followed by the addition of 1% turkey RBCs in PBS (Gibco; Life Technologies Corporation, Grand Island, NE, USA; Cat: 10010023) in each well. Plates were incubated for 30 min at RT to distinguish between agglutinated and non-agglutinated wells prior imaging. For hemagglutination assays measuring the reversibility of zinc-mediated hemagglutination inhibition, samples were incubated at room temperature for a minute, hour, or a day after zinc exposure and prior to incubation with turkey RBCs. Hemagglutination units (HAU) of samples incubated with zinc ions were normalized to the HAU of each IAV stock used in the absence of zinc ions.

### 2.4. Passaging Assays

Viruses diluted to a multiplicity of infection (MOI) of 0.002 for a 6-well plate of MDCK cells were incubated with 2 or 10 mM zinc chloride for 1 h at room temperature. Then zinc ions were chelated by adding equimolar EDTA disodium salt dissolved in water pH 8.0 (Lab Chem, Zelienople, PA, USA; Cat: LC137501) and the treated viruses added to MDCK cells. After 48 h, supernatants were collected, frozen and stored as described previously [41]. Supernatants were titrated using plaque assay and used to passage the viruses at an MOI of 0.002 6 times. Prior to every infection, diluted viruses were treated with 2 or 10 mM zinc chloride as described above.

### 2.5. Reverse-Transcription PCR

RNA extraction from Trizol was performed as described previously. Spike RNA was purchased from IDT DNA. Isolated RNA was reverse transcribed using SuperScript III and a primer binding to the 3′ end of the RNA. qPCR was performed as described previously. The Spike RNA had the sequence (5′ → 3′):

AGUAGAAACAAGGCGGUAGGCGCUGUCCUUUAUCCAGACAACCAUUACCUGUCCACACAAUCUGCCCUUUCGAAAGAUCCCAACGAAAAGAGAGACCACAUGGUCCUUCCUGCUUUUGCU.

### 2.6. Data Analysis and Statistics

Data were analyzed in Graphpad Prism 8 using one-way ANOVA with multiple corrections. A Mann–Whitney U test was used to compare the recovery percentages between conditions.

## 3. Results

### 3.1. Zinc and Copper Ions Reduce IAV Titers

**Figure 1:**
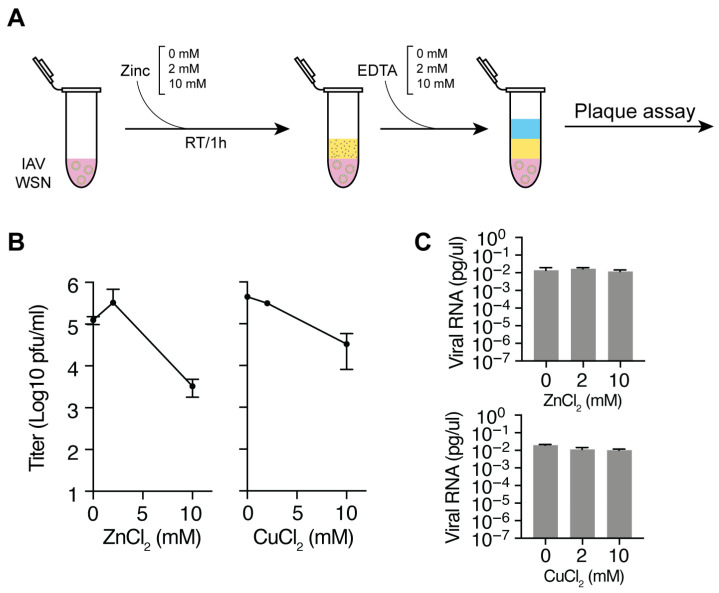
IAV is inactivated by zinc and copper ions. (**A**) Schematic of the experimental approach for inactivating IAV with zinc or copper ions and neutralization of the zinc or copper ions with EDTA. (**B**) IAV titers after exposure to zinc or copper chloride and neutralization with EDTA as measured on MDCK cells. (**C**) NA segment RT-qPCR analysis after exposure of IAV to zinc or copper chloride and neutralization with EDTA. Data points represent mean ± SD (*n* = 3).

Copper and zinc containing surfaces, fabrics and particles have been reported to inactivate IAV strains and SARS-CoV-2. To confirm that copper and zinc ions can inactivate these two RNA viruses in our setup, we incubated a fixed titer of influenza A/WSN/33 (H1N1) with 0–10 mM of zinc or copper chloride. After 60 min, the reactions were stopped with an equimolar amount of EDTA and subsequently diluted for virus titer determination by plaque assay (Figure 1A). In line with previous observations [33], we found a concentration-dependent reduction in both the IAV titer in the presence of zinc and copper chloride (Figure 1B). In addition, we tested the effect of a mixture of copper and zinc on the IAV and found an increased reduction in virus titer compared to zinc or copper chloride alone (Figure 1B). We previously showed that EDTA alone does not affect the virus titer [33].

It was previously shown that IAV and SARS-CoV-2 exposure to zinc ions does not result in viral RNA release [33]. To confirm this result, we treated zinc, copper or dually treated IAV with RNase and extracted RNA after a 1 h incubation. An external non-viral control RNA was added as loading control during the RNA extraction. Next, we performed reverse transcription using an internal or 3′ terminal segment 6 primer and quantified the viral cDNA levels using qPCR. No effect was observed on IAV RNA levels after incubation with zinc or copper chloride (Figure 1C). Together, these results confirm that zinc and copper ions can inactivate influenza A/WSN/33 (H1N1) in a concentration-dependent manner in our setup and that they do not affect the integrity of the viral particle, in line with our previous observations [33].

### 3.2. Zinc-Mediated Inhibition of IAV Hemagglutination

Given the relatively low toxicity of zinc, we focused on further characterizing the zinc ions-mediated inactivation of IAV. Building on the above and previous observation that zinc ions leave the viral RNA intact [33], we hypothesized that zinc ions directly affected the IAV HA protein and thereby impaired host cell receptor binding. A well-established assay for testing HA functionality is the hemagglutination assay, which relies on a change in RBC sedimentation upon the binding of IAV HA proteins to the sialic acid receptors on RBCs. While this assay does not provide an absolute quantitative measurement of sialic acid binding, it can be used to measure the relative change in the interaction between HA and sialic acid receptors expressed on RBCs. To test whether zinc ions directly affect the interaction between HA and host sialic acid receptors, we measured the sialic acid receptor binding ability of A/WSN/33 (H1N1) after exposure to zinc ions using turkey RBCs.

**Figure 2:**
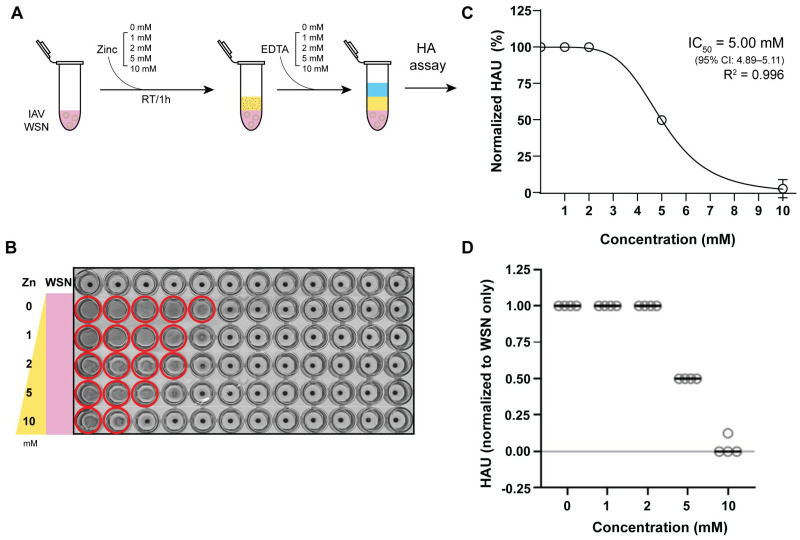
Zinc-mediated inhibition of IAV hemagglutination occurs in a concentration-dependent manner. (**A**) Experimental design illustrating the evaluation of zinc-mediated IAV hemagglutination inhibition across various zinc ion concentrations and subsequent neutralization with equimolar EDTA. (**B**) Assessment of sialic acid (SA) binding by IAV virions as measured by the hemagglutination of 1% turkey red blood cells (RBCs) after a 30 min incubation of IAV with serially diluted zinc ions. Red circles indicate hemagglutination. (**C**) Hemagglutination units (HAU) of four independent IAV treatments (**D**) with zinc ions, normalized to the untreated IAV samples. Nonlinear regression analysis was performed to determine the IC_50_ of zinc-mediated hemagglutination inhibition using a variable slope model. The best-fit IC_50_ was 5.00 mM (95% CI: 4.89–5.11 mM) and the goodness-of-fit analysis yielded an R^2^ value of 0.996. Data points represent mean ± SD (*n* = 4).

We first incubated 1 HA unit (HAU) (1.18 × 10^6^ pfu) IAV strain A/WSN/33 (H1N1) with different concentrations of ZnCl_2_ in DMEM supplemented with 2% of FBS. After an hour of incubation at room temperature (Figure 2A), zinc ions were chelated with an equimolar amount of EDTA to prevent zinc-mediated cytotoxic effects on turkey RBCs during the hemagglutination assay (Figure 2B). ZnCl_2_ and EDTA-treated IAV samples were next mixed with 1% RBCs and incubated for 30 min to allow the RBCs to settle in round-bottom plates. As shown in Figure 2C, the hemagglutination levels of IAV samples treated with 1 or 2 mM ZnCl_2_ were comparable to RBCs incubated with untreated IAV. RBCs incubated with IAV treated with 5 mM ZnCl_2_ or 10 mM ZnCl_2_ showed a decrease in hemagglutination. The IC_50_ for zinc-mediated inhibition of hemagglutination was 5.00 mM (Figure 2C). These findings align with our previous results showing a significant reduction in IAV titers after 1 h of incubation with 10 mM ZnCl_2_. These observations also suggest that zinc ions inhibit IAV HA from binding to sialic acid receptors in a concentration-dependent manner, as higher ZnCl_2_ concentrations significantly reduced hemagglutination.

### 3.3. Zinc Reduces IAV Hemagglutination Within 1 Minute of Incubation

**Figure 3:**
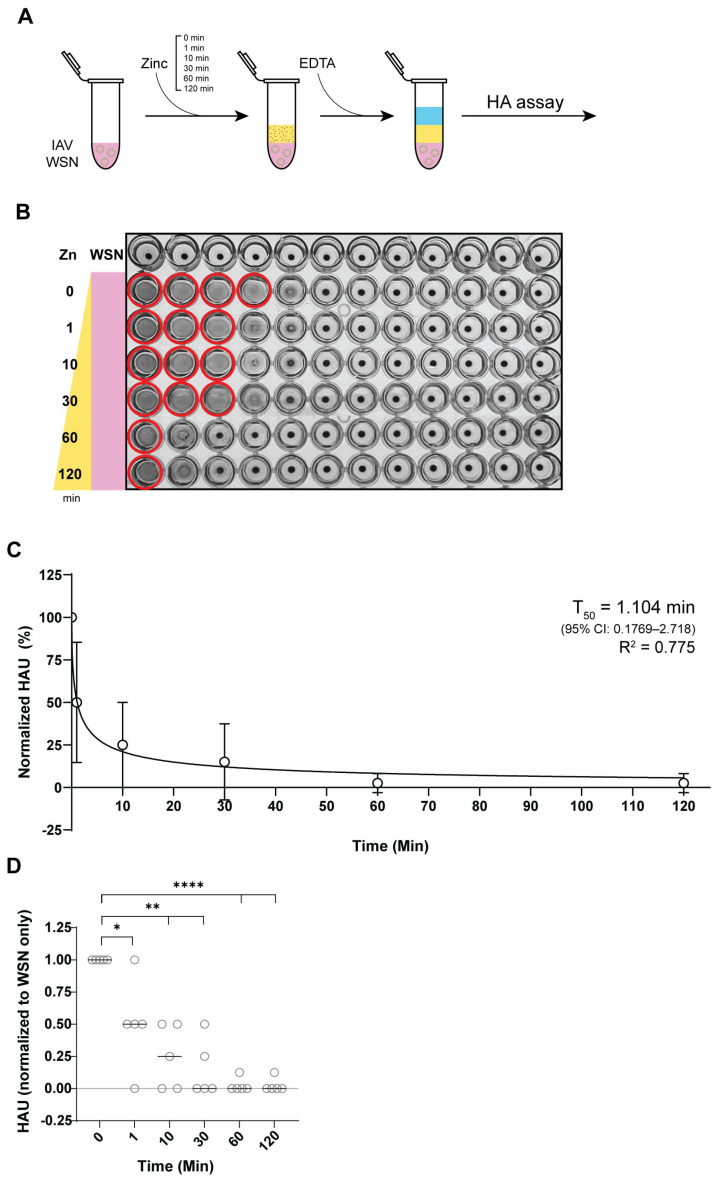
Zinc ions rapidly inhibit IAV hemagglutination within 1 min of exposure. (**A**) Experimental design for evaluating the time-dependent inhibition of IAV hemagglutination by zinc ions and subsequent neutralization with 10 mM EDTA. (**B**) Assessment of sialic acid (SA) binding of IAV virions based on the hemagglutination of 1% turkey RBCs after incubation of the IAV samples with 10 mM zinc chloride for 0–120 min. Zinc ions were neutralized with equimolar EDTA before incubation with turkey RBCs. Solid red circles indicate hemagglutination. (**C**) Hemagglutination units (HAU) of IAV samples treated with zinc ions, normalized to the untreated IAV control. Nonlinear regression analysis was performed to determine the T_50_ of zinc-mediated hemagglutination inhibition using a variable slope model. The best-fit T_50_ was 1.104 min (95% CI: 0.1769–2.718 mM) and the goodness-of-fit analysis yielded an R^2^ value of 0.7749. (**D**) Same dataset as in panel C, displayed with individual data points from five independent biological replicates to illustrate variability. Statistical comparisons were performed using Welch’s *t*-test between each time point and the 0 min control. Significant differences are indicated with asterisks, * *p* ≤ 0.05, ** *p* ≤ 0.01, **** *p* ≤ 0.0001.

To assess the time required for zinc-mediated hemagglutination inhibition, we examined how different ZnCl_2_ incubation periods affected hemagglutination reduction. Samples were incubated with 10 mM ZnCl_2_ for 1, 10, 30, 60, or 120 min (Figure 3A) and each reaction was terminated using 10 mM EDTA. To minimize variation in ETDA incubation time, zinc exposure of IAV was staggered, with exposure initiated at different time points, followed by simultaneous termination with 10 mM EDTA. All hemagglutination assays were performed in parallel to reduce confounding factors.

Analysis of the hemagglutination level showed that a 1 min exposure to 10 mM ZnCl_2_ was sufficient to significantly reduce hemagglutination levels compared to the untreated IAV control (Figure 3B). The extent of inhibition was comparable between samples exposed to zinc ions for 1 to 30 min. Zinc-mediated inhibition was greatest when IAV samples were exposed to 10 mM zinc ions for 1 h, with no additional reduction observed with longer incubation (Figure 3C,D). Using an inhibition curve fit, we calculated the 50% inhibition time to be 1.104 min, demonstrating that rapid zinc-mediated HA inactivation occurs within a short time frame (Figure 3C). These findings indicate that zinc ions exert their antiviral effect on HA rapidly, with significant inhibition occurring within the first minute of exposure and reaching maximal inhibition within an hour, reinforcing the potential for zinc-embedded interventions.

### 3.4. Zinc-Mediated Reduction in Influenza a Virus Hemagglutination Retained for 24 h

**Figure 4:**
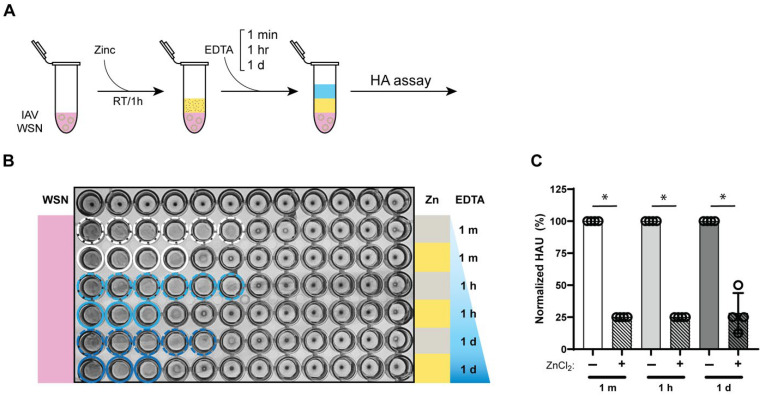
Zinc-mediated HA inactivation is sustained for at least 24 h. (**A**) Experimental design for evaluating the persistence of zinc-mediated IAV hemagglutination inhibition for 1 min, 1 h, and 24 h after zinc ion neutralization with equimolar EDTA. (**B**) Assessment of sialic acid (SA) binding by measuring hemagglutination of 1% turkey RBCs with IAV samples incubated at room temperature for the specified times with zinc (yellow) or no zinc (gray) followed by zinc neutralization with EDTA. Colored solid or dashed circles indicate hemagglutination. (**C**) Hemagglutination units (HAU) of IAV samples treated with zinc ions, normalized to the untreated IAV samples that were incubated for the same time as the zinc ion treated condition. A Mann–Whitney U test was used to compare the recovery percentages between conditions, * *p* ≤ 0.05.

To assess the durability of zinc-mediated hemagglutination inhibition, we examined how long the antiviral effect persisted. After treating IAV with 10 mM ZnCl_2_ for 1 h and subsequent zinc chelation, the samples were further incubated at room temperature for 1 min, 1 h, or 24 h before their hemagglutination levels were assessed (Figure 4A). Control IAV samples were initially incubated for 1 h without zinc ions and then for a further 1 min, 1 h or 24 h at identical room temperature incubation conditions. As shown in Figure 4B,C, the hemagglutination inhibition achieved after 1 h of zinc exposure was maintained for the entire 24 h period, with comparable levels of inhibition observed. Since free zinc ions were chelated with EDTA immediately after the 1 h incubation, no further zinc-mediated inactivation could occur during the subsequent 24 h period. Overall, these findings indicate that zinc-mediated hemagglutination inhibition remains effective and stable for at least 24 h, suggesting that the antiviral properties of zinc ions persist over an extended period of time, even after zinc ions are removed.

### 3.5. Zinc-Mediated Hemagglutination Inhibition Is Maintained Across Different Temperatures

PPE to protect against infectious pathogens may be used in different or fluctuating environments. For instance, face masks experience higher temperatures and higher moisture content than biohazard coveralls [42]. Even different parts of a PPE may experience different conditions. The inner surface of a face mask, which is in direct contact with exhaled breath and facial heat, reaches an average temperature of 34.7–35.0 °C, while the outer surface is exposed to an average temperature of 34.2–34.9 °C. Given that temperature can influence chemical interactions, protein conformations, and ion solubility, we sought to determine whether the antiviral activity of zinc ions against IAV is affected by temperature.

**Figure 5:**
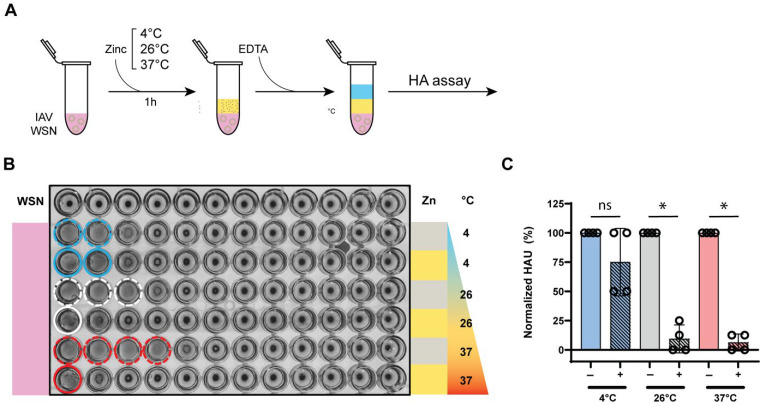
Zinc-mediated HA inactivation remains effective across various temperatures. (**A**) Experimental design for evaluating the zinc-mediated IAV hemagglutination inhibition at 4 °C, 26 °C, and 37 °C. (**B**) Assessment of sialic acid (SA) binding by measuring hemagglutination of 1% turkey RBCs with IAV samples incubated at different temperatures in presence (yellow) or absence (gray) of zinc ions for 1 h followed by zinc neutralization with EDTA. Colored solid and dashed circles indicate hemagglutination. (**C**) Hemagglutination units (HAU) of IAV samples treated with zinc ions, normalized to the untreated IAV samples that were incubated at the same temperature as the zinc ion treated condition. A Mann–Whitney U test was used to compare the recovery percentages between conditions, * *p* ≤ 0.05, ns, non significant.

To explore the impact of temperature on IAV inactivation by zinc ions, we incubated IAV with 10 mM ZnCl_2_ at 4 °C, 26 °C, or 37 °C for one hour and chelated the zinc ions with equimolar EDTA at the end of the exposure time (Figure 5A). The impact of temperature on IAV inactivation was then assessed using hemagglutination assays, which were performed in RT, consistent with our prior experiments. IAV samples incubated at 37 °C exhibited zinc-mediated HA inactivation at levels comparable to those incubated at 26 °C (Figure 5B). Samples incubated with zinc at 4 °C showed greater variation in hemagglutination inhibition (Figure 5C), but this variability may stem from reduced solubility of ZnCl_2_ in lower temperatures, as zinc solubility is affected by temperature [43,44]. Overall, zinc-mediated IAV inactivation was significant at both 26 °C and 37 °C, indicating that the antiviral effects of zinc are robust across a range of physiologically and environmentally relevant temperatures.

### 3.6. Virus Passaging After Zinc Exposure Does Not Lead to Resistance Emergence

Exposure of IAV to antivirals, such as favipiravir, can lead to resistance emergence [41,45]. To investigate whether exposure to zinc ions could also lead to resistance, IAV were treated with 2 or 10 mM zinc ions for 1 h and then used to infect MDCK cells at an MOI of 0.002. After 48 h, the supernatants were collected, the viruses titrated, and the procedure repeated. Overall, we treated and passaged IAV 6 times at the two different zinc ion concentrations, but did not observe an increase in titer or decrease in zinc sensitivity (Table 1), suggesting that repeated exposure to zinc ions up to 6 times does not lead to resistance emergence in IAV.

## 4. Discussion

The ongoing challenges posed by emerging RNA viruses, such as SARS-CoV-2 and avian IAV, underscore the need for the continued development of antiviral PPE. In particular, there is a growing interest in the application of metal ions, such as silver, copper, and zinc, into nanoparticles and fabrics as a means of providing virucidal activity [46,47]. However, the precise mechanism through which these metal ions exert viral inactivation is still poorly understood.

In this study, we investigated the antiviral properties of zinc ions against IAV. In particular, we focused on their ability to inhibit the host receptor binding function of the IAV HA protein, which mediates viral receptor binding and entry into the host cell. Our findings indicate that zinc ions can inhibit hemagglutination in a concentration-dependent manner (Figure 2). Moreover, our observations indicate that zinc ions can inhibit hemagglutination quickly, with 50% inhibition observed in ~1 min of exposure. The maximum inhibition was observed within 1 h (Figure 3). In addition, we observed that the antiviral effect of zinc ions was retained for up to 24 h, indicating a sustained or even irreversible mechanism of action (Figure 4), and that zinc-mediated IAV inactivation occurred over a wide temperature range, with significant IAV inactivation at 26 °C and 37 °C (Figure 5).

How does IAV activation occur in the presence of zinc ions? Based on our observations (Figure 1) and previous work by Gopal et al. [33], which showed no detectable viral RNA release following zinc treatment, we infer that the viral envelope and/or the M1 scaffold remains intact after zinc exposure. Our data suggest that instead HA function is impaired by zinc ions. This is in agreement with a previous study that demonstrated that HA can interact with zinc ions and undergo conformational rearrangements analogous to changes in HA trimers under acidic conditions after zinc exposure [40]. In addition, this study indicated that zinc ions can stimulate multimerization or aggregation of recombinant HA in vitro. Together with our observations, the current literature thus indicates that zinc ions trigger a conformational change and/or aggregation of HA, which in turn impairs receptor binding and hemagglutination. It remains possible that other viral surface proteins, such as neuraminidase or M2, are also affected by zinc ions and we suggest that their stability and function is measured in future studies.

The HA assay exclusively reports on HA-mediated receptor binding, and our data only supports the conclusion that zinc disrupts the interaction between HA and sialic acid receptors. Importantly, because HA-mediated receptor binding is the initial and essential step for viral entry, targeting this interaction represents a meaningful and effective approach for antiviral application. Our observations suggest that zinc ions directly affect HA protein function, reducing its ability to bind to host cell receptors. This finding aligns with recent reports from Seok et al., which showed that divalent zinc ions bind to Glu68 and His137 on the HA1 domain and induce structural changes in HA similar to those triggered by acidic pH. In cleaved HA, exposure to low pH leads to irreversible structural changes that facilitate membrane fusion [40]. The precursor HA0 also experiences reversible yet extensive conformational rearrangements at acidic pH, characterized by a wider molecular envelope and dilated and rotated membrane-distal domains. Given that the zinc-binding residues are located near the receptor binding domain (RBD), it is plausible that zinc-induced perturbations of the HA1 head region could interfere with receptor binding, even in uncleaved HA0. Further structural research will be needed to determine the extent and nature of zinc-mediated conformational changes in uncleaved HA within intact virions.

The critical role of PPE in protecting healthcare workers and mitigating SARS-CoV-2 transmission was clearly demonstrated during the COVID-19 pandemic [48,49,50]. However, the pandemic also highlighted significant challenges, such as the risks associated with incorrect PPE use and the incorrect disposal of PPE, which may lead to exposure to virions trapped within the fabric. Our findings, which demonstrate a rapid and sustained inhibition of IAV HA function by zinc ions, highlight the potential of zinc-embedded fabrics as a PPE material with immediate virucidal activity that is long-lasting and active across a range of temperatures. By preventing virus entry into host cells, this fabric could significantly reduce the risk of infection at its earliest stage, which would be of value in high-risk environments, such as healthcare settings. Moreover, the sustained protection provided by zinc-embedded PPE would not only stop the virus from initiating replication but would also improve safety during and after use.

Overall, these findings reinforce the potential utility of zinc-based antiviral strategies in PPE applications, particularly in settings where temperature fluctuations occur. The observed consistency in zinc-mediated HA inactivation at higher temperatures supports the feasibility of zinc ion-embedded materials as self-cleaning antiviral barriers for face masks and other protective equipment. With avian IAV spillovers becoming more frequent, especially among individuals who are in close contact with livestock, effective virus-inactivating PPE could be a key measure in reducing the occurrence of zoonotic transmission and preventing the next pandemic. While we here based our work on the A/WSN/33 (H1N1) strain and additional studies are required to confirm that zinc can offer similar protection efficiencies against avian influenza strains, we think that our work advances our understanding of the virucidal properties of zinc and hope that it will inspire further research.

## Figures and Tables

**Table 1:** IAV titers after treatment with zinc ions and 48 h growth (first two columns) or titer after exposure to zinc to measure sensitivity (last two columns).

Passage	Mean Titer After 2 mM Zinc Passage (pfu/mL)	Titer After 10 mM Zinc Passage (pfu/mL)	Titer of 2 mM Passage After 10 mM Zinc Challenge (pfu/mL)	Mean Titer of 10 mM Passage After 10 mM Zinc Challenge (pfu/mL)
Untreated	7.26 × 10^7^	7.26 × 10^7^	1.00 × 10^5^ (input)	1.00 × 10^5^ (input)
1	7.83 × 10^6^	8.91 × 10^5^	7.29 × 10^4^	5.13 × 10^3^
2	6.37 × 10^7^	3.11 × 10^6^	8.98 × 10^4^	4.96 × 10^3^
3	9.41 × 10^6^	2.83 × 10^6^	4.73 × 10^4^	1.58 × 10^3^
4	7.17 × 10^6^	6.97 × 10^5^	9.33 × 10^4^	5.73 × 10^3^
5	4.52 × 10^7^	8.22 × 10^5^	7.11 × 10^4^	8.92 × 10^2^
6	2.92 × 10^7^	2.09 × 10^6^	5.54 × 10^4^	4.66 × 10^3^

## Data Availability

Data are contained within the article.
